# Isolation of *Burkholderia* sp. HQB-1, A Promising Biocontrol Bacteria to Protect Banana Against Fusarium Wilt Through Phenazine-1-Carboxylic Acid Secretion

**DOI:** 10.3389/fmicb.2020.605152

**Published:** 2020-12-10

**Authors:** Zhizhou Xu, Mingyuan Wang, Jinpeng Du, Ting Huang, Jianfu Liu, Tao Dong, Yinglong Chen

**Affiliations:** ^1^Research Center of Horticultural Science and Engineering, Huaqiao University, Xiamen, China; ^2^College of Plant Protection, Nanjing Agricultural University, Nanjing, China; ^3^Institute of Fruit Tree Research, Guangdong Academy of Agricultural Sciences, Guangzhou, China; ^4^UWA Institute of Agriculture, UWA School of Agriculture and Environment, The University of Western Australia, Perth, WA, Australia

**Keywords:** antifungal activity, *Burkholderia*, banana *Fusarium* wilt, LC-Q-TOF-MS, phenazine-1-carboxylic acid

## Abstract

Fusarium wilt is a devastating soil-borne fungal disease caused by *Fusarium oxysporum* f.sp. *cubense* (Foc). In recent years, some antifungal bacteria have been applied for the prevention and biocontrol of pathogenic fungi. In our study, a bacterial strain HQB-1, isolated from banana rhizosphere soil, was cultured for investigation. It showed broad-spectrum antifungal activities against representative phytopathogenic fungi including *Fusarium oxysporum*, *Colletotrichum gloeosporioides*, *Botrytis cinerea*, and *Curvularia fallax*. The strain HQB-1 was identified as *Burkholderia* sp. by morphological, physiological, and biochemical examinations, confirmed by 16S rRNA gene sequence analysis. Among the metabolites produced by the strain, we identified an antifungal compound which was identified phenazine-1-carboxylic acid (PCA) (C_13_H_8_N_2_O_2_) through ultraviolet, liquid chromatography quadrupole-time of flight mass spectrometer, and nuclear magnetic response. Furthermore, PCA exhibited the lowest minimum inhibitory concentration (MIC) against *F. oxysporum* (1.56 μg/ml) and yielded the highest MIC against *C. gloeosporioides*. Pot experiments showed that application of 5 μg/ml or more of PCA efficiently controlled banana wilt and promoted the growth of banana plants. These results suggested that *Burkholderia* sp. HQB-1, as an important microbial resource of PCA, could be a promising biological agent against wilt diseases and promoting banana growth.

## Introduction

Banana (*Musa* spp.), is one of the most important fruit crops, widely cultivated in tropical and subtropical areas, providing staple food for over 400 million people (Dale et al., 2017). With the increase in exportation, banana becomes an important source for foreign exchange in China (Wang B. et al., 2013). However, in recent years, Fusarium wilt, also known as Panama disease, has become one of the most destructive soil-borne diseases threatening banana production worldwide (Davis et al., 1996; Ploetz, 2005; Fourie et al., 2011). Fusarium wilt is caused by *Fusarium oxysporum* f. sp. *cubense* (Foc) with the Tropical Race 4 (Foc TR4) causing the most severe damage among the four races of this pathogen. It has been demonstrated that the pathogen Foc TR4 can infect banana roots, penetrate into the root xylem, spread into the rhizome and pseudostem xylem within several days after infection (Warman and Aitken, 2018), and eventually cause the occlusion of vascular bundle and the blocking of water and nutrients transportation (Li et al., 2017). Once Foc TR4 was introduced to or detected in banana orchard, it is quite difficult to eradicate. Because the chlamydospores of Foc TR4 can survive for a long period of time in soil (Viljoen, 2002), it is becoming one of the most crucial limiting factors in banana production (O’Donnell et al., 1998; Lin et al., 2009). Therefore, it is necessary and imperative to efficiently control the occurrence and spread of Foc TR4 in banana.

Traditionally, chemical pesticide is considered to be a simple and direct approach to control Fusarium wilt (Fuchs et al., 1999; Getha et al., 2005). However, chemical residues not only provided unsatisfactory control levels but also lead to severe environmental pollutions and induce the development of resistant strains (Kanini et al., 2013). Though resistant breeding cultivars have been studied and widely used for a long time, polyploidy, low reproductive fertility and complete sterility of banana are the main reasons for the limited success of the breeding program (Shepherd, 1987). Crop rotation slowly reduces pathogen density in the soil but its effectiveness is restricted once the disease outbreak occurred (Chellemi et al., 2016). Recently, biological control, especially the application of biocontrol microorganisms, has been widely studied due to its advantage as an economically and environmentally friendly method (Leeman et al., 1996). However, the available microorganisms for biocontrol of plant diseases may also lead to inconsistent performance and poor activity in the soil environment (Saravanan et al., 2003). Thus, the isolation and screening of highly efficient and broad-spectrum antagonistic microorganisms are the key for the development of biocontrol agents. New bioformulation technologies like nanotechnologies, formulation of microbial consortia and/or their metabolites need to be developed to improve their efficacy and deepen the knowledge of biocontrol microorganisms (Bubici et al., 2019).

The genus *Burkholderia* has become increasingly important during the past several decades because of its capability in producing abundant secondary metabolites with antimicrobial, insecticidal, herbicidal, or growth-promotion traits (Jeong et al., 2003; Singh et al., 2013; Kunakom and Eustáquio, 2019). Several *Burkholderia* strains like *B. vietnamiensis*, *B. ambifaria*, and *B. pyrrocinia* have been reported to be potential and efficient biological control agents (Mahenthiralingam et al., 2008). In fact, the key mechanism for the antagonistic effects of *Burkholderia* against plant pathogens is the production of antimicrobial secondary metabolites (Schmidt et al., 2010; Eberl and Vandamme, 2016). Among them, cepacin, phenazine, and pyrrolnitrin have been shown to play roles as antibiotics in controlling plant diseases (Parker et al., 1984; Cartwright et al., 1995; Xu et al., 2013). Siderophores produced by *Burkholderia* not only exhibit survival competition with pathogens but also promote the growth of the plant (Jiang et al., 2008). However, a large number of new natural compounds produced by *Burkholderia* is still unknown, and the isolation, purification, and identification of these bio-functional natural products, especially as antimicrobials, need to be further investigated.

In this study, we screened and isolated a broad-spectrum antagonistic bacterial strain HQB-1 from the rhizosphere soil of banana plants. The taxonomic status of HQB-1 was determined as *Burkholderia* sp. according to the 16S rRNA gene sequence analysis combined with morphological, physiological, and biochemical characteristics. Moreover, the antifungal component phenazine-1-carboxylic acid (PCA) produced by *Burkholderia* sp. HQB-1 was separated and identified by column chromatography, ultraviolet (UV), liquid chromatography quadrupole-time of flight mass spectrometer (LC-Q-TOF-MS), and nuclear magnetic response (NMR). The bio-controlling effect of PCA was further investigated via pot experiments. The aim of this study was to analyze the antifungal metabolites from potential beneficial microbial resources and to identify the main efficient compound that could be used to prevent Fusarium wilt in banana.

## Materials and Methods

### Soil Sample Collection, Isolation of Fusarium Wilt Antagonistic Bacteria and Antifungal Activity

Twelve rhizosphere soil samples including clay and silt loam and were collected from healthy plants found in a pathogen-infested banana orchard, which has been planted banana for 15 years, at Changtai County, Zhangzhou City, Fujian Province, China (24.65°N, 117.72°E) as described by Chen et al. (2013). The plants were gently removed from the farmland, the loose soil was removed by shaking, and the soil attached to the roots was collected by a brush. The soil samples were placed into clean, dry, and sterile polythene bags, transported to the laboratory, and stored at 4°C until further analysis.

The samples were suspended in 0.85% of NaCl and spread on Luria-Bertani (LB) agar. After incubation at 30°C for 2 days, bacterial colonies with different morphological characteristics were selected and re-streaked on fresh LB agar until the homogeneous colonies appeared. Fifty-six isolated strains were further sub-cultured in LB broth on a rotary shaker. For long-term storage of bacterial strains, 50% (v/v) glycerol solution was added to the liquid culture for freezing at −80°C.

Several important phytopathogenic fungi were chosen and used in the present study, included *Fusarium oxysporum* f. sp. *cubense* Tropical Race 4 (Foc TR4, ATCC 76255), *Colletotrichum gloeosporioides* (ATCC 16330), *Botrytis cinerea* (ATCC 11542), and *Curvularia fallax* (ATCC 38579). These strains were provided by the Institute of Tropical Bioscience and Biotechnology, China Academy of Tropical Agricultural Sciences, Haikou, China. The antifungal activity of isolated strains was determined according to Reyes-Chilpa et al. (1997) and Chen et al. (2018) with slight modifications. Briefly, a fungal plug (Φ = 6 mm) was inoculated in the center of a plate filled with potato dextrose agar (PDA) medium; Each isolated bacterium was seeded with a sterile stick at a distance of 2.5 cm from the fungi. A fungal disk in the center of a plate without seeding isolated bacterium was served as a control. After incubation at 28°C for 5 days, the inhibition zone was recorded by measuring the distance between the edge of the fungal mycelium and the bacterial colony. The percentage inhibition of mycelial radial growth was calculated by the following formula:

Percent mycelial inhibition = $\frac{\text{A}-\text{B}}{\text{A}}\times 100$, where A is the mean colony diameter for the control, and B is the mean colony for the treatment (Nimaichand et al., 2015).

### Morphology, Physiological, and Biochemical Tests of Antagonistic Bacteria

The morphological, physiological, and biochemical characteristics tests of the strain HQB-1 were further analyzed including: Gram-staining, temperature and pH range for growth, presence of pigment, catalase activity, oxidative or fermentative reaction, nitrate reduction, hydrolysis of casein, starch, Tween 80 and gelatin, activity of urease and pyruvate deaminase, tolerance to sodium chloride, production of indole, reaction of H_2_S, Methyl Red-staining, and Voges-Proskauer tests. All these characteristics were then compared with the standard description of Bergey’s Manual of Determinative Bacteriology (Buchanan and Gibbons, 1984).

To obtain scanning electron microscope (SEM) image of HQB-1, a 1 cm^2^ transverse section of the bacterial strain on LB agar plate was obtained and fixed in 2.5% glutaraldehyde buffer at 4°C for 3 h. Then the bacterial cells were washed by phosphate-buffered saline (PBS) for three times and fixed in 1% osmic acid at 25°C for 5 h. After washing, the cells were dehydrated with 50, 70, 95, and 100% ethanol, respectively, for critical point drying and platinum-coating using an ion-sputter coater. The morphology of the specimen was observed at an opening voltage of 5 kV using a field-emission scanning electron microscopy (S-4800IIESEM, Hitachi, Japan).

### 16S rRNA Gene Sequencing and Phylogenetic Analysis

The strain HQB-1 was cultured in LB broth at 28°C in an orbital shaker for 12 h. The genomic DNA of the strain was extracted using the bacterial genomic DNA extraction kit (Takara, Beijing, China). The 16S rRNA sequences were amplified by PCR with the universal primers 27F (5′-GAGAGTTTGATCCTGGCTCAG-3′) and 1492R (5′-CTACGGCTACCTTGTTACGA-3′) (Gupta et al., 2014). The PCR conditions were as follows: initial denaturation at 94°C for 5 min, 30 cycles of 94°C for 45 s, 58°C for 60 s, and 72°C for 90 s, and a final extension at 72°C for 10 min. The PCR products were purified by Quick Gel Extraction Kit (TransGen Biotech, Beijing, China) and sequenced by Sangon Biotech (Shanghai, China). The 16S rRNA gene sequence was submitted to the GenBank database. The sequences were analyzed by a global alignment algorithm implemented in the EzBioCloud database^1^ (Kim et al., 2012; Yoon et al., 2017) and blasted with the sequences of known species in NCBI database^2^. After multiple alignments of sequences via CLUSTAL_X (Thompson et al., 1997), the phylogenetic analysis was performed using the neighbor-joining method (Saitou and Nei, 1987) with MEGA version 6.0 (Tamura et al., 2007).

### Fermentation of HQB-1 and Separation of Antifungal Components

Forty liters of LB broth with HQB-1 were grown in a 200 L fermentation tank.at 30°C for 4 days. At the end of fermentation, the cultural broth with HQB-1 was centrifuged at 10,000 × *g* for 20 min, and an equal volume of ethyl acetate (EA) was added into the cultural supernatant. This procedure was repeated three times. The EA layers were combined and the solvent was evaporated under vacuum at 40°C using a rotary evaporator for obtaining the final crude extract with approximately 36 g. The crude EA extract was then subjected to macroporous resin (XAD-16, Amberlite, United States) column chromatography and eluted with different (30, 60, and 100%) aqueous MeOH to yield three fractions (Fractions 1–3). The fractions were tested in plates for their antifungal activity, where a Foc TR4 mycelial plug (6 mm in diameter) was inoculated into 10 ml potato dextrose broth (PDB) per dish with 25 μg/ml of each fraction or DMSO (0.1% in sterile water, negative control). The most active fraction (Fraction 3) with visible inhibition was then passed through a silica gel (200 meshes) column (Sigma-Aldrich, Shanghai, China) and eluted with different ratios of methanol and chloroform (subfractions 1–3 were eluted with methanol and chloroform at the ratio of 1:3, 1:6, and 1:9, respectively).

### Purification and Chemical Analysis of the Main Antifungal Component

The subfractions were also tested in the bioassays as described above, and the most active fraction was subjected to the analysis of the UV absorption spectrum (Shimadzu UV-2450, Japan). Liquid chromatographic separation was conducted in a reverse-phased C18 analytical column (Zorbax RRHD Eclipse Plus, 2.1 × 50 mm, 1.8 μm, Agilent, United States). A syringe pump delivering 5 μl was adjusted by the direct loop injection method (Hu et al., 2005). The mobile phase A was water with 0.1% acetic acid (v/v) and the mobile phase B was methanol. The chromatographic separation was held at the initial mobile phase composition (5% methanol) constant for 0.1 min, followed by a linear gradient to 95% methanol for 6 min, then decreased to 5% in 6–8 min, and followed by a one min post-run time after each analysis. All samples were performed on high performance liquid chromatography (HPLC) using a solvent containing 30% mobile phase A and 70% mobile phase B for 2 min, operated at a flow rate of 0.4 ml/min and the UV absorption at 365 nm. The HPLC-separated samples were then analyzed by quadrupole-time of flight mass spectrometer (TOF-MS) (Agilent1290-6545, United States). Electrospray ionization (ESI) was operated in negative ionization mode with other parameters used as following: capillary voltage was 4.0 kV; the temperature of dry gas (N_2_) was 200°C; the flow rate of dry gas was 9 L/h at 35 psig spray pressure; the temperature of sheath was 350°C; the flow rate of sheath was 10 L/h, and the ion scanning range was m/z 100∼1100. The collision energy was set at 10∼40 eV for the MS/MS analysis. The mass data of the molecular ions were processed through the Mass Hunter Qualitative Analysis (version B.07.00) software.

Meanwhile, the bioactive subfraction was solved in deuterated chloroform (CDCl_3,_ δ = 77.0 ppm) at the concentration of 10 mg/ml for the ^1^H-NMR and ^13^C-NMR experiments with a 5 mm probe by a Bruker spectrometer (500 MHz/Advance III, Advance Digital, Bruker, Germany). The spectra were obtained at 30°C using tetramethylsilane (TMS) as the internal standard. The NMR spectra were analyzed by MestReNova (version 6.1.1-6384) software.

### Determination of the Minimum Inhibitory Concentration (MIC) of PCA

A standardized 96-well microdilution broth assay (Wedge and Kuhajek, 1998) was used to evaluate the antifungal activity of the pure compound from *Burkholderia* sp. HQB-1.

The MICs of phenazine-1-carboxylic acid (PCA) from the cultural broth of the strain HQB-1 against the fungal pathogens including *F. oxysporum*, *C. gloeosporioides*, *B. cinerea*, and *C. fallax*. Different concentrations of PCA (50.0, 25.0, 12.5, 6.25, 3.13, 1.56, 0.78, 0.39 and 0.20 μg/ml) were prepared by using 2-fold serial dilutions for minimum inhibitory concentration (MIC) test. The lowest concentration of PCA that inhibited fungal growth was recorded as the MIC. The concentration of the inoculated fungal pathogens was 1 × 10^6^ CFU/ml. Carbendazim was used as a positive control and 0.1% DMSO as a negative control. The 96-well plates were covered with a plastic lid and incubated at 25°C for 72 h. The absorbance was measured at 620 nm by a microplate photometer (BioTek, BioTek Instrument Co., United States). Mean absorbance and standard errors were used to evaluate fungal growth after 72 h incubation (Wang X. et al., 2013).

### Biological Assays

Biological assays were carried out in July–September 2019 at the greenhouse in Huaqiao University, Xiamen. Pathogen suspensions were prepared as follows: a 6 mm diameters agar disk containing 7-day-old pathogen was added to a flask containing PDB. The flask was incubated at 28°C, 170 rpm for 7 days, and then the suspension was filtered through four layers of sterilized gauze. The conidial suspension was determined using a biological microscope (Nikon, Japan) and adjusted with distilled water to give a final concentration of 1.0 × 10^6^ CFU/ml for further use.

The soil used in this study was a mixture of peat and perlite (2:1, v/v) and sterilized at 120°C for 2 h. Three to four true leaves old banana (*Musa acuminata* L.) AAA group, cv. *Cavendish* seedlings (Shishengyuan Biological Technology Co., Ltd., Gaozhou, China) were transplanted in plastic pots (8 cm × 8 cm, with one plant standing in one cup) with 80 g soil for pot experiment. The fungal pathogen *F. oxysporum* Tropical Race 4 with a concentration of 1.0 × 10^6^ CFU/ml was inoculated into the potting substrates 5 days before PCA treatments. Six treatment group were conducted as follows: (1) CK1 (negative control, non-inoculated Foc TR4 and application of 0.1% DMSO); (2) CK2 (positive control, inoculated Foc TR4 and application with sterile water); (3) PCA 1 μg/ml; (4) PCA 5 μg/ml; (5) PCA 25 μg/ml; (6) PCA 50 μg/ml. Each PCA treatment group was watered weekly with 5 ml PCA at the concentration of 1, 5, 25, or 50 μg/ml mixed in sterile water. Seven plants were included for each treatment, and the experiment was repeated three times. The banana seedlings were placed on a greenhouse bench with natural light for 60 days and watered regularly.

The physiological indexes of banana seedlings treated for 60 days were determined, including root length, plant height, stem diameter, leaf width and dry weight. The disease symptoms on each plant were recorded and rated in five classes from 0 to 4 according to Mak et al. (2004). The disease index (DI) was calculated as DI (%) = Σ (Class × Number of plants in that class)/(4 × Total number of assessed plants) × 100. Biocontrol efficiency (BE) was calculated using the following formula: BE (%) = (DI in sterile water controlled - DI in treatments)/DI in sterile water controlled × 100.

### Statistical Analysis

Statistical analysis was performed using SPSS 19. The difference among treatments was determined based on one-way analysis of variance (ANOVA), and means were subjected to Duncan’s multiple range test with significance set at *P* < 0.05.

## Results

### Isolation of the Strain HQB-1

**FIGURE 1:**
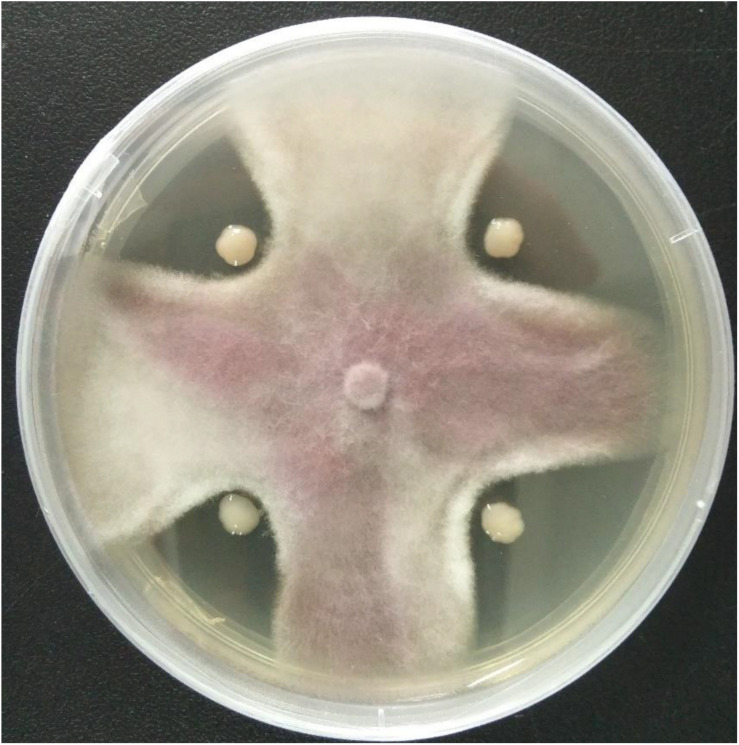
Antagonistic activity of the strain HQB-1 against *Fusarium oxysporum* Tropical Race 4 (Foc TR4). The co-cultivation HQB-1 and Foc TR4 grown on PDA medium at 28°C for 5 days.

A total of 11 bacterial isolates with 33.3 to 56.7% antagonistic effect was isolated from the soil samples in banana orchard. Among them, the isolate HQB-1 showed a more significant and stronger antagonistic activity against Foc TR4 on the plate (Figure 1) and exhibited a broad-spectrum antifungal activity against other plant pathogens. The growth inhibition of *F. oxysporum*, *C. gloeosporioides, B. cinerea*, and *C. fallax* was 56.7, 36.7, 52.4, and 52.2% respectively, after 5-day cultivation with HQB-1 (Table 1).

**TABLE 1:** Antifungal activities of strain HQB-1 against various pathogens.

Pathogenic fungi (strain#)	Mycelial inhibition (%)	Antifungal activity
*Fusarium oxysporum* (ATCC 76255)	56.7 ± 0.64a	++++
*Colletotrichum gloeosporioides* (ATCC 16330)	36.7 ± 1.15b	+++
*Botrytis cinerea* (ATCC 11542)	52.4 ± 0.83a	++++
*Curvularia fallax (*ATCC *38579)*	52.2 ± 0.68c	++++

*Data are means ± SD (*n* = 3). Data of mycelial inhibition followed by different letters are significantly different at *p* < 0.05 level according to Duncan’s multiple range test. The result of antifungal activity was expressed as the distance between the fungal pathogen and the area of antagonist growth after 5 days. - no inhibition zone; + (very weak), 0–5 mm; + + (weak), 5–10 mm; +++ (moderate), 10–15 mm; ++++ (strong), 15–20 mm; +++++ (very strong), >20 mm.*

### Morphological, Physiological, and Biochemical Identification of the Strain HQB-1

**FIGURE 2:**
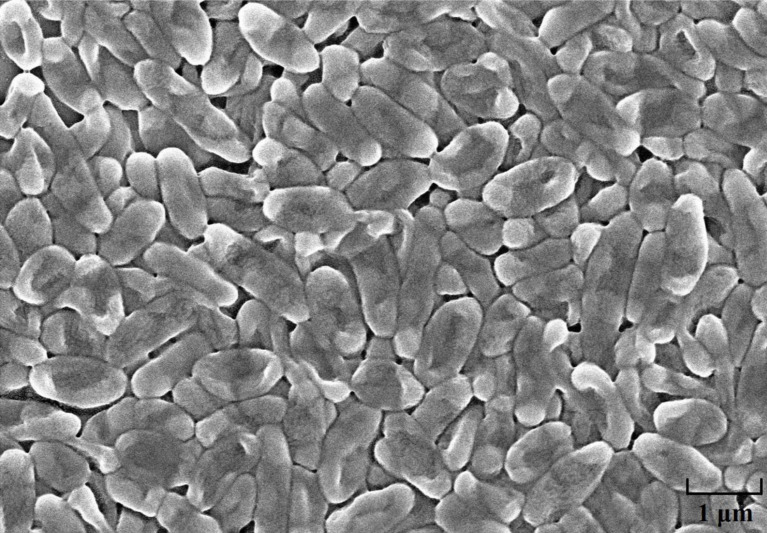
Scanning electron microscope image of HQB-1. Morphological characters of *Burkholderia* sp. HQB-1 viewed using SEM. Scale bar means 1 μm in the image.

The strain HQB-1 was slightly yellow, shining, and smooth in colony appearance on the nutrient agar (NA) plate. The biochemical tests indicated that it is a gram-negative and rod-shaped bacterium. The shape of the strain HQB-1 was straight rod, and the size was (1.2–2.5) μm × (0.5–1.0) μm (Figure 2) under scanning electron microscope (SEM). The tests of catalase, methyl red, oxidative or fermentative acid production from carbohydrates, liquefaction of gelatin, phenylalanine deaminase and indole were positive; and the Voges-Proskauer tests, tests for the presence of pigment, hydrolysis of starch, urease, Tween 80, H_2_S production, and creatine were negative. Besides, the strain HQB-1 can grow at a temperature range of 18–39°C.

### 16S rRNA Gene Sequencing and Phylogenetic Analysis of the Strain HQB-1

**FIGURE 3:**
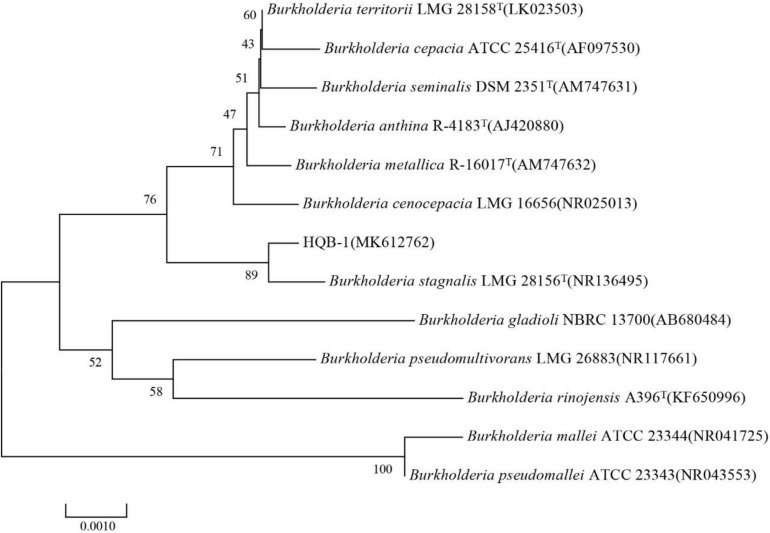
Phylogenetic tree of the strain HQB-1 based on 16S rRNA gene sequence analysis. The bootstrap values (%) presented at the branches were calculated from 1,000 replications. The numbers in parentheses are the GenBank accession numbers. The scale bar 0.001 represents 2 nucleotide substitutions per 1,000 nucleotides. T means type strain.

The 16S rRNA gene sequence (1471 bp) was submitted to GenBank with accession number MK612762. The sequence exhibited 99.7% similarity with *Burkholderia stagnalis* LMG 28156^*T*^ (NR136495) by comparison against the EzBioCloud database and the BLASTn database in GenBank. After multiple alignments of sequences via CLUSTAL_X, a phylogenetic tree based on the neighbor-joining method using MEGA version 6.0 software demonstrated that HQB-1 fell into the cluster comprised of *Burkholderia* species (Figure 3). Therefore, based on morphological, physiological, and biochemical characteristics as well as the 16S rRNA gene sequence analysis, the strain HQB-1 is supposed to be affiliated to the genus *Burkholderia*.

### Separation and Purification of the Main Active Antifungal Component

**FIGURE 4:**
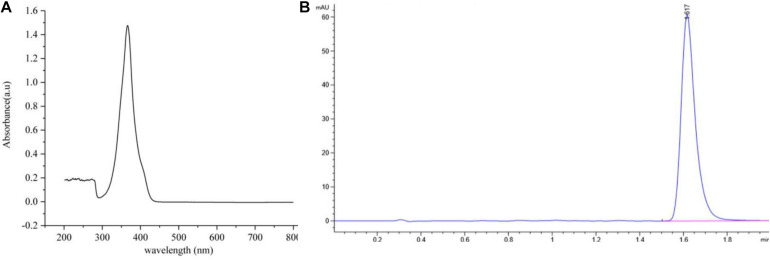
The ultraviolet and HPLC data of the main active antifungal component produced by *Burkholderia* sp. HQB-1. **(A)** The UV absorption of the main active subfraction at a wavelength of 0-800 nm, the peak means the maximum absorption at the wavelength of 365 nm; **(B)** Chromatogram of the main active component conducted in C18 column with detection at 365 nm. The flow rate was set at 0.4 ml⋅min^–1^ and the presence of a single peak at 1.617 min indicated that the high purity compound. Absorbance × Retention Time.

Antifungal activity assays showed that the most active antifungal compound was eluted with 100% MeOH by macroporous resin column (Fraction 3) (Supplementary Figure 1). The subfraction with the strongest antifungal activity eluted in methanol and chloroform (1:9, v/v) was discovered using silica gel column chromatography (Supplementary Table 1). The solvent was slowly evaporated at room temperature to obtain greenish-yellow crystals. The chemical analysis of this active compound was performed by using UV and HPLC. The UV spectrum data (Figure 4A) showed a strong absorption and a broad peak at 365 nm, which indicated the presence of a phenazine moiety in the molecule. Then the active compound was subjected to HPLC with a C18 column, and a single peak with a retention time of 1.617 min under the 365 nm was detected (Figure 4B).

### Structure Identification of the Main Active Antifungal Component

**FIGURE 5:**
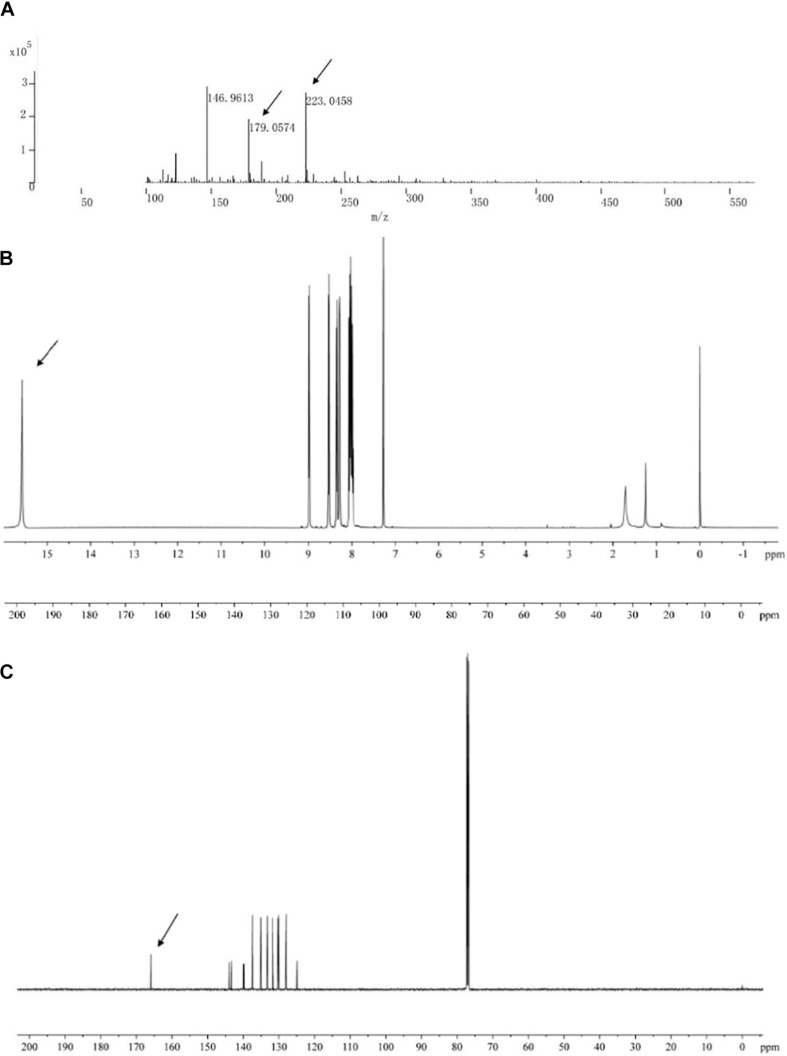
Chemical analysis of the main active antifungal component produced by *Burkholderia* sp. HQB-1. **(A)** liquid chromatography quadrupole-time of flight mass spectrometer (LC-Q-TOF-MS). The ion peaks pointed by the arrows indicate the presence of carbon and proton MS data of m/z 179.0574 and m/z 223.0458, respectively. **(B,C)** ^1^H-NMR and ^13^C-NMR spectrum. The spectral data (δ in ppm, J in Hz) were measured at 30°C with CD_3_Cl as the solvent. The tetramethylsilane (TMS) was used as internal standard.

The main active antifungal component was then subjected to Q-TOF-MS and NMR. The MS spectra showed a molecular ion peak at m/z 223.0458 and the molecular formula as C_13_H_8_N_2_O_2_ was deduced (Figure 5A). The theoretical MS (Q-TOF) value of C_13_H_8_N_2_O_2_ ([M-H]^–^) was calculated for mass 223.0513 and in this study mass 223.0512 was found. The MS/MS spectra of the component displayed a fragment ion peak at m/z 179.0574, which may be due to the loss of COOH. The major fragment ion at m/z 179 was close to the relative molecular mass of a phenazine ring.

**FIGURE 6:**
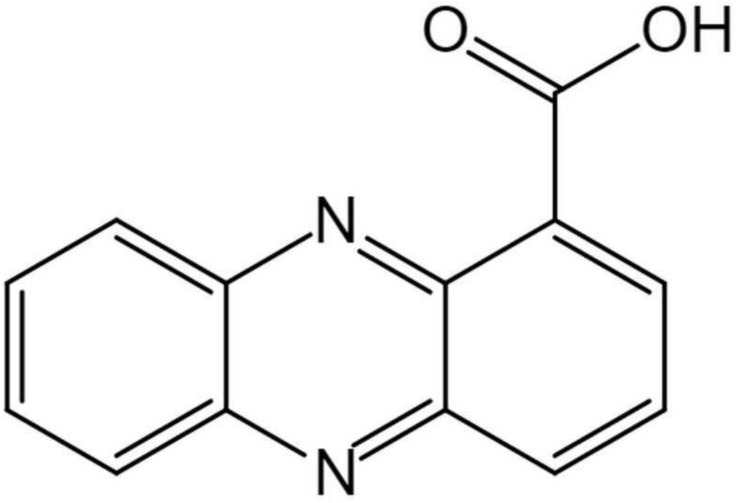
Chemical structure of (phenazine-1-carboxylic acid) PCA isolated produced by *Burkholderia* sp. HQB-1.

The structure of the active compound was further identified by ^1^H-NMR and ^13^C-NMR spectroscopy. There were eight and thirteen well-resolved signals available in the ^1^H-NMR and ^13^C-NMR spectrum, respectively. Seven peaks in the 7.30–9.00 ppm region were assumed to be aromatic protons and the peak at 15.58 ppm was assigned to the carboxylic acid proton (Figure 5B). In the ^13^C-NMR spectrum, twelve peaks in 124.91–144.07 ppm were assigned to be aromatic carbons, which indicated a symmetrical structure for the molecule. The peak at 165.93 ppm clearly indicated the presence of carbonyl carbon (Figure 5C). Meanwhile, the NMR spectra were compared with Scifinder^3^ and SDBS information database^4^, which indicated the target molecule as phenazine-1-carboxylic acid (PCA) (C_13_H_8_N_2_O_2_) (Figure 6).

### MIC of PCA

According to the 96-well microtiter assay, the MIC values of purified PCA against *F. oxysporum*, *C. gloeosporioides*, *B. cinerea*, and *C. fallax* were 1.56, 6.13, 1.56, and 3.13, respectively (Table 2), which were lower than carbendazim treatments used as positive control. The lowest MIC of PCA was 1.56 μg/ml against *F. oxysporum* and *B. cinerea*. Whereas, the highest MIC of PCA was 6.13 μg/ml against *C. gloeosporioides*. The negative control (0.1% DMSO treated) had no inhibitory effect on the tested pathogenic fungi.

**TABLE 2:** Values of minimum inhibitory concentration (MIC) of (phenazine-1-carboxylic acid) PCA against pathogenic fungi.

Pathogenic fungi	MIC of PCA (μg/ml)	MIC of carbendazim (μg/ml)	MIC of DMSO (μg/ml)
*Fusarium oxysporum* (ATCC 76255)	≥1.56	≥3.13	–
*Colletotrichum gloeosporioides* (ATCC 16330)	≥6.13	≥12.5	–
*Botrytis cinerea* (ATCC 11542)	≥1.56	≥6.25	–
*Curvularia fallax (*ATCC 38579*)*	≥3.13	≥6.25	–

*–, no inhibitory effect.*

### Effect of the Active Antifungal Component in Pot Experiments

**FIGURE 7:**
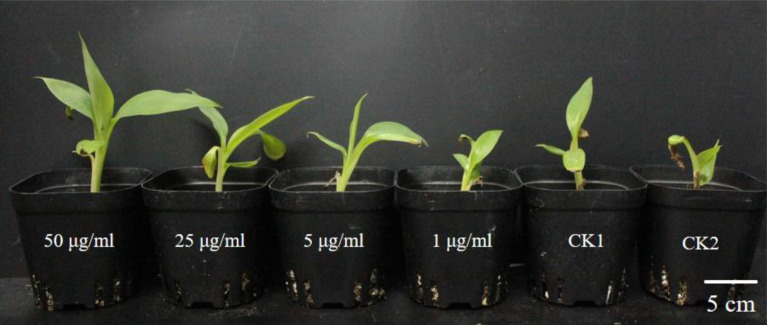
Biocontrol by treatments with different concentrations of phenazine-1-carboxylic acid (PCA) on banana plants. Plants were grown for 60 days and watered weekly using water mixed with 5 ml of PCA at different concentrations. From left to right: banana plants were treated by 50, 25, 5, and 1 μg/ml PCA, respectively; CK1, negative control, non-inoculated Foc TR4 and application of 0.1% DMSO; CK2, positive control, inoculated Foc TR4 and application with sterile water. Scale bar means 5 cm in the image.

The disease incidence measured for various treatments with different PCA concentrations (1, 5, and 25 μg/ml) were 40.8 ± 1.55, 19.1 ± 2.08, and 14.29% ± 1.84, respectively (Table 3). Meanwhile, the biocontrol efficiency was 51.2 ± 1.68, 77.2 ± 1.75, and 82.9% ± 1.43 respectively. PCA treatment at the concentration of 50 μg/ml completely inhibited Fusarium wilt in banana plants demonstrating strong antifungal activity against *F. oxysporum in vivo* (Figure 7). Furthermore, PCA treatments significantly promoted the growth of banana plants including root length, plant height, stem girth, leaf width, and dry weight compared with the control (Table 4). When the concentration of PCA reached more than 5 μg/ml, there was no significant difference in root length of banana plants between treatments. However, when the concentration of PCA reached more than 25 μg/ml, the difference in the growth of plant height was insignificant compared with DMSO treatment.

**TABLE 3:** The biocontrol effects of (phenazine-1-carboxylic acid (PCA) against banana Fusarium wilt.

PCA (μg/ml)	1	5	25	50	CK1	CK2
DI/%	40.8 ± 1.55b	19.1 ± 1.08bc	14.3 ± 1.84d	0e	15.7 ± 1.52ab	100a
BE/%	51.2 ± 1.68cd	77.2 ± 1.75c	82.9 ± 1.43b	100a	–	–

*DI, disease index; BE, Biocontrol efficiency. CK1, negative control, non-inoculated Foc TR4 and application of 0.1% DMSO; CK2, positive control, inoculated Foc TR4 and application with sterile water. –, not detected. Data in the table are means ± SD (*n* = 7). For each parameter, mean data followed by different letters are significantly different at the *P* < 0.05 level by the least significant difference (LSD) test.*

**TABLE 4:** Effects of different concentrations of (phenazine-1-carboxylic acid) PCA treatments on the growth of banana plants.

Plant items	PCA (μg/ml)				CK1	CK2
	1.00	5.00	25.00	50.00		
Root length (cm)	2.62 ± 0.02d	3.87 ± 0.30c	3.91 ± 0.34b	4.47 ± 0.26a	3.53 ± 0.22bc	0.83 ± 0.35e
Plant height (cm)	10.13 ± 1.32c	12.47 ± 1.55ab	12.53 ± 0.85ab	13.90 ± 0.36a	12.57 ± 0.25ab	8.33 ± 1.10cd
Stem diameter (cm)	1.10 ± 0.10bcd	1.30 ± 0.10abc	1.47 ± 0.15a	1.37 ± 0.06a	1.33 ± 0.12ab	0.87 ± 0.23d
Leaf width (cm)	2.20 ± 0.61ab	2.57 ± 0.32ab	2.90 ± 0.46a	2.57 ± 0.60ab	2.45 ± 0.05ab	1.40 ± 0.30c
Dry weight (g)	1.00 ± 0.29d	1.42 ± 0.16c	1.87 ± 0.22a	1.55 ± 0.38ab	1.36 ± 0.11bcd	0.52 ± 0.15e

*CK1, negative control, non-inoculated Foc TR4 and application of 0.1% DMSO; CK2, positive control, inoculated Foc TR4 and application with sterile water. Data in the table are means ± SD (*n* = 7). For each parameter, mean data followed by different letters are significantly different at the *P* < 0.05 level by the least significant difference (LSD) test.*

## Discussion

Soil-borne plant diseases, especially Fusarium wilt, cause a significant decline of the production in banana field (Ploetz, 2005). Biological control is considered to be a novel and efficient way to prevent and inhibit banana Fusarium wilt. The screening of antagonistic bacteria from soil microorganisms in fields with banana Fusarium wilt is of great practical significance in controlling this disease (Duan et al., 2020). In the soil bacteria community of healthy banana plants, the *Burkholderia* may be one of the most abundant genera that contribute to suppressing Fusarium wilt (Shen et al., 2014). It was well demonstrated that most of the *Burkholderia* sp. strains isolated from the rhizosphere of healthy plants have been used for controlling such plant disease and promoting the growth of the plant (Parke and Gurian-Sherman, 2001; Compant et al., 2008; Gu et al., 2009). In our study, the *Burkholderia* sp. strain HQB-1 was isolated and screened from banana rhizosphere soil. It exhibited an excellent antagonistic effect (56.7 ± 0.64%) against *F. oxysporum* and a broad-spectrum antifungal activity, which indicated that *Burkholderia* sp. HQB-1 has the potential to be a promising candidate against banana Fusarium wilt.

The genus *Burkholderia* was used as a biological agent against phytopathogenic fungi mainly due to its production of plentiful metabolites with antimicrobial activity (Hu and Young, 1998). In this study, to identify the potential antagonistic mechanisms of *Burkholderia* sp. HQB-1, we investigated the effect of the secondary metabolites secreted by this strain on inhibiting the mycelial growth of Foc TR4. The HQB-1 cultural broth was filtered through the 0.22 μm microfiltration membranes for salting-out and dialysis, whereas, the antimicrobial tests indicated that neither the proteins nor the enzymes in the HQB-1 metabolites possessed antifungal activity. The siderophore produced by the strain HQB-1 was determined by the chrome azurol S (CAS) method (Bultreys et al., 2001), but the quantitative tests showed that HQB-1 was not high-yield in siderophore (data not shown). However, the crude HQB-1 ethyl acetate extract possessed antifungal activity against *F. oxysporum*, which suggested that the secondary metabolites secreted from HQB-1 might contain antimicrobials. A greenish-yellow crystal was further purified by column macroporous resin and silica gel column chromatography, which exhibited a strong antifungal activity against Foc TR4 mycelial plug in plates. The antifungal component showed a strong absorbance peak at 365 nm (Figure 4A) with UV spectral analysis, which indicated that the antifungal component might be a molecule bearing phenazine moiety (Hu et al., 2005). Furthermore, the ^1^H-NMR spectrum (Figure 5B) revealed the presence of 8 aromatic protons and an ABCD pattern in the aromatic ring, the characteristics of which are typically found in phenazine derivatives. The ^13^C-NMR spectrum (Figure 5C) revealed the presence 13 aromatic carbons that indicated a highly symmetrical structure for the target molecule (Gurusiddaiah et al., 1986). Both of the peaks at 15.58 ppm in ^1^H-NMR and at 165.93 ppm in the ^13^C-NMR spectrum demonstrated the presence of a carboxyl. Additionally, by compared with the NMR spectrum data in the SDBS information database, the chemical structure of the antifungal component was confirmed as phenazine-1-carboxylic acid (PCA) (Figure 6; Abraham et al., 2015).

Gurusiddaiah et al. (1986) found that the MIC values of PCA isolated from *Pseudomonas fluorescens* strain 2-79 against *F. oxysporum* was 25–30 μg/ml using the agar dilution method. PCA at 3.12 μg/ml reduced the mycelial growth of *B. cinerea* by 50%, and inhibited the formation of exopolysaccharide (Simionato et al., 2017). Zheng et al. (2019) reported that the MIC value of crude extracts of *Streptomyces* sp. FJAT-31547 against *F. oxysporum* was 6.250 μg/ml. In this study, the MIC values of PCA against plant pathogenic fungi were 1.563–6.13 μg/ml according to the results from 96-well microtiter assay (Table 2). The lowest MIC was 1.56 μg/ml against *F. oxysporum*, which indicated that PCA was more efficient than carbendazim. Thus, we proposed that PCA produced by *Burkholderia* sp. HQB-1 might be a key factor for the bacterial broad-spectrum antifungal activity against plant phytopathogenic fungi.

Phenazine-1-carboxylic acid has been reported to play an important role in inhibiting *F. oxysporum* in diverse crops such as chickpea, tomato, and wheat (Anjaiah et al., 1998; Chin-A-Woeng et al., 1998; Pastor et al., 2016). It seemed to be a more contributor to biocontrol *Fusarium oxysporum* than pyrrolnitrin in *Pseudomonas fluorescens* (Upadhyay and Srivastava, 2011). PCA is the main active component of the newly registered green biopesticide “Shenqinmycin,” and its broad-spectrum inhibition of plant fungal pathogens of has been applied (Jin et al., 2015). The Shenqinmycin suspension with a concentration as low as 1% can prevent rice sheath blight, pepper blight, and cucumber seedling damping-off (Xu, 2013). However, the biocontrol effect of PCA against banana Fusarium wilt has not been well studied. In the present study, we investigated not only the antifungal activity of the PCA produced by HQB-1 against several phytopathogenic fungi was effective both *in vitro* and *in vivo*. Most of the banana plants in the current study could be efficiently protected from *F. oxysporum* infection, thus preventing Fusarium wilt, by 5 μg/ml PCA (BE, 77.2 ± 1.75%). Treatment with PCA at 500 μg/ml could protect most of the pepper plants from *Phytophthora* infection, and it also showed significant protective effect against anthracnose development on cucumber leaves (Lee et al., 2003). However, the mechanism of the antifungal effect of PCA remains unclear. Previous research reported that phenazine compounds are involved in inhibiting RNA synthesis and DNA binding (Morrison and Marley, 1976; Palchykovska et al., 2008), thereby interfering with redox balance and inducing the generation of reactive oxygen species (ROS) which eventually causes fungi death (Morales et al., 2010). Moreover, the phenazines may also have important physiological functions within phenazine-producing bacteria themselves. For example, it has been reported that the phenazine derivative pyocyanin leads to decreased levels of NADH in *Pseudomonas aeruginosa* grown under anoxic condition (Xu et al., 2013). We also found that proper concentrations (5–25 μg/ml) of PCA could increase the height and biomass, especially the root length of the banana plants compare with CK2 group (Table 4). This indicated the potential of PCA in facilitating water uptake, nutrient mobilization and growth promotion in banana plants. Thus, we propose that PCA might be critical for the broad-spectrum antifungal activity of *Burkholderia* sp. HQB-1 and it possesses a strong biocontrol effect on banana plants. The study of the antagonistic mechanism of PCA against *F. oxysporum* in banana and its practical application needs to be further investigation.

## Data Availability Statement

The raw data supporting the conclusions of this article will be made available by the authors, without undue reservation, to any qualified researcher.

## Author Contributions

ZX and MW conceived the study and prepared the manuscript. ZX, JD, and TH performed the experiments and analyzed the data. MW, JL, TD, and YC supervised and provided the suggestion of the research work. YC helped to revise the manuscript. All authors have read and approved the manuscript before submission.

## Conflict of Interest

The authors declare that the research was conducted in the absence of any commercial or financial relationships that could be construed as a potential conflict of interest.

## Footnotes

1 http://eztaxon-e.ezbiocloud.net/

2 https://blast.ncbi.nlm.nih.gov/Blast.cgi

3 http://scifinder.cas.org

4 https://sdbs.db.aist.go.jp/
