# Supplementary material for: Isolation of Burkholderia sp. HQB-1, A Promising Biocontrol Bacteria to Protect Banana Against Fusarium Wilt Through Phenazine-1-Carboxylic Acid Secretion
Source: Front Microbiol. 2020 Dec 10;11:605152. doi: 10.3389/fmicb.2020.605152 (PMC7758292; doi:10.3389/fmicb.2020.605152)
Supplement: Supplementary file 1 [file Data_Sheet_1.doc]

Supplementary material

**Table 1** The antifungal activity of subfractions against *Fusarium oxysporum* Tropical Race 4 (Foc TR4).

| Subfractions | 1 | 2 | 3 | CK |
| --- | --- | --- | --- | --- |
| Antifungal effect/% | 4.49±1.58b | 30.68±2.76b | 80.51±2.58a | - |

-, no antifungal effect. Subfractions 1-3 represent the elution of methanol and chloroform at the ratio of 1:3, 1:6, and 1:9 respectively. CK, positive control, inoculated Foc TR4 and application with sterile water. Data in the table are means ± SD (n=3). Different lowercase letters in the same column show values that are significantly different at the P < 0.05 level by the least significant difference (LSD) test.


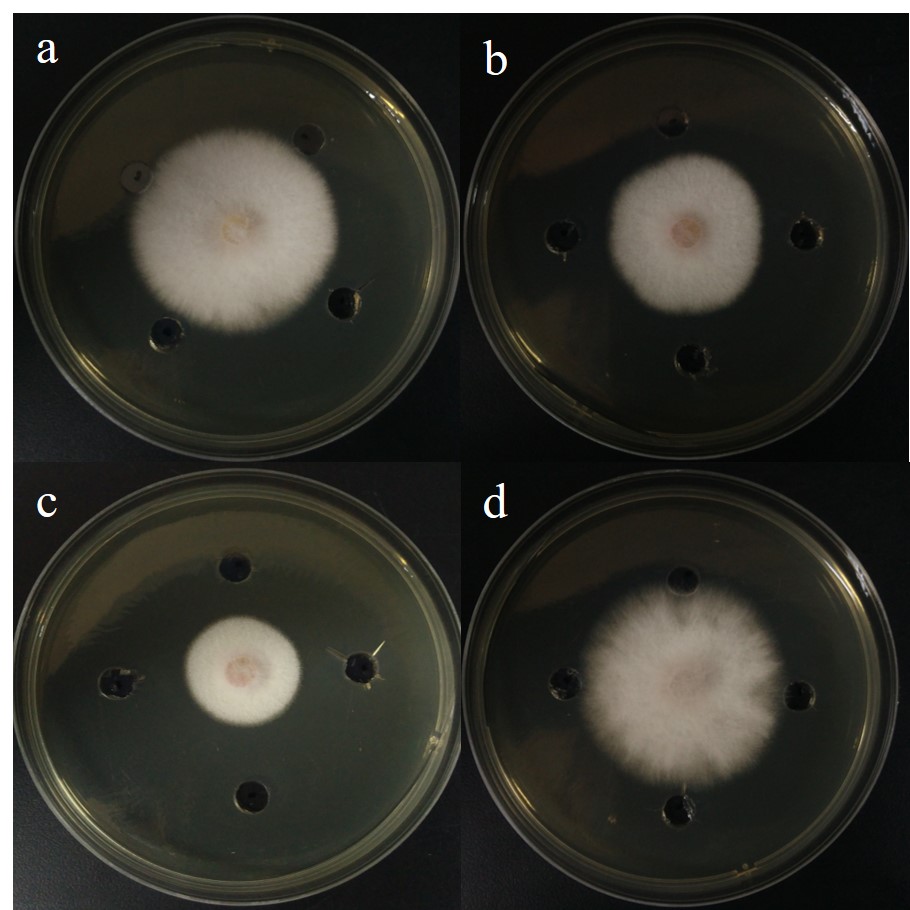


Figure 1. The antifungal activity against *Fusarium oxysporum* Tropical Race 4 (Foc TR4) of fractions eluted by gradient methanol. (a-c) Fractions eluted by 30%, 60%, and 100% methanol respectively. Fractions (5 μl) were added into the agar well at the distance of 2.5 cm away from Foc TR4. (d) Negative control. 5 μl of sterile water was applied to the agar well.
